# NMDA-Receptor Activation but Not Ion Flux Is Required for Amyloid-Beta Induced Synaptic Depression

**DOI:** 10.1371/journal.pone.0065350

**Published:** 2013-06-04

**Authors:** Albert Tamburri, Anthony Dudilot, Sara Licea, Catherine Bourgeois, Jannic Boehm

**Affiliations:** Département de Physiologie, Groupe de Recherche sur le Système Nerveux Central, Université de Montréal, Montréal, Québec, Canada; Thomas Jefferson University, United States of America

## Abstract

Alzheimer disease is characterized by a gradual decrease of synaptic function and, ultimately, by neuronal loss. There is considerable evidence supporting the involvement of oligomeric amyloid-beta (Aβ) in the etiology of Alzheimer’s disease. Historically, AD research has mainly focused on the long-term changes caused by Aβ rather than analyzing its immediate effects. Here we show that acute perfusion of hippocampal slice cultures with oligomeric Aβ depresses synaptic transmission within 20 minutes. This depression is dependent on synaptic stimulation and the activation of NMDA-receptors, but not on NMDA-receptor mediated ion flux. It, therefore, appears that Aβ dependent synaptic depression is mediated through a use-dependent metabotropic-like mechanism of the NMDA-receptor, but does not involve NMDA-receptor mediated synaptic transmission, i.e. it is independent of calcium flux through the NMDA-receptor.

## Introduction

Alzheimer’s disease (AD) is the most common neurodegenerative disorder of modern society. It affects the elderly and causes impaired memory formation and loss of higher cognitive functions [1]. Brains of AD patients usually contain two pathological protein agglomerates: extracellular plaques composed of amyloid-beta (Aβ) peptides and intracellular neurofibrillary tangles composed of hyper-phosphorylated tau protein.

Through studies conducted on human patients and animal AD models, it is now widely accepted that Aβ is implicated in the cause of AD [2]. Elevated levels of this peptide disrupt glutamatergic synapses in the hippocampus and cortex [3]–[5]. Neurons exposed to Aβ have impaired long-term potentiation [6], [7] and increased depression of synaptic transmission, most likely by activating pathways involved in synaptic long-term depression [8], [9]. Interestingly, deficits in synaptic transmission appear before the formation of amyloid plaques in AD transgenic mouse models [10]. Furthermore, the magnitude of memory and learning disabilities in AD patients does not correlate well with the amyloid plaque burden [11]. These findings suggest that plaque formation may not be the primary cause for synaptic failure in early AD. Consequently, over the last years oligomeric Aβ has been identified as one of the main factors for the changes found in synaptic transmission in AD [12], [13].

Most experimental models currently used in AD research chronically incubate neuronal cultures with Aβ, by either exposing neurons to oligomeric Aβ for several hours or by using animal models overexpressing Aβ. This approach, although generally accepted for mimicking the conditions occurring during AD, makes it challenging to analyze the acute effects of Aβ on synaptic transmission. Given that neurons can regulate synaptic strength with mechanisms such as homeostatic plasticity, it becomes difficult to dissect primary from secondary effects on synaptic transmission by Aβ, i.e. the neuron can counter-regulate the synaptic insult caused by Aβ. To address the immediate effects of Aβ on synaptic transmission, we used an acute perfusion protocol of Aβ oligomers in hippocampal slice cultures. Our results show that acutely applied Aβ oligomers promote synaptic depression in hippocampal CA1 pyramidal neurons within 15 min. This depression is mediated by glutamate dependent activation of NMDA receptors but appears to be independent of NMDA-receptor mediated ion flux. In summary, we propose that Aβ oligomers promote synaptic depression through NMDA-receptors acting in a metabotropic rather than ionotropic fashion.

## Results

### Acute Aβ Perfusion Leads to NMDA-receptor Dependent Synaptic Depression

Previous studies in which we participated have shown that the overexpression of the amyloid precursor protein (APP) in hippocampal neurons leads to synaptic depression in organotypic hippocampal slice cultures [14]. This depression was dependent on the activation of NMDA-receptors and further analysis revealed that APP expression activates a LTD-like intracellular signaling cascade. Given that the overexpression of APP leads to a chronic exposure of neurons to Aβ, we wanted to analyze this depression in a more acute and more controlled experimental setup.

**Figure 1:**
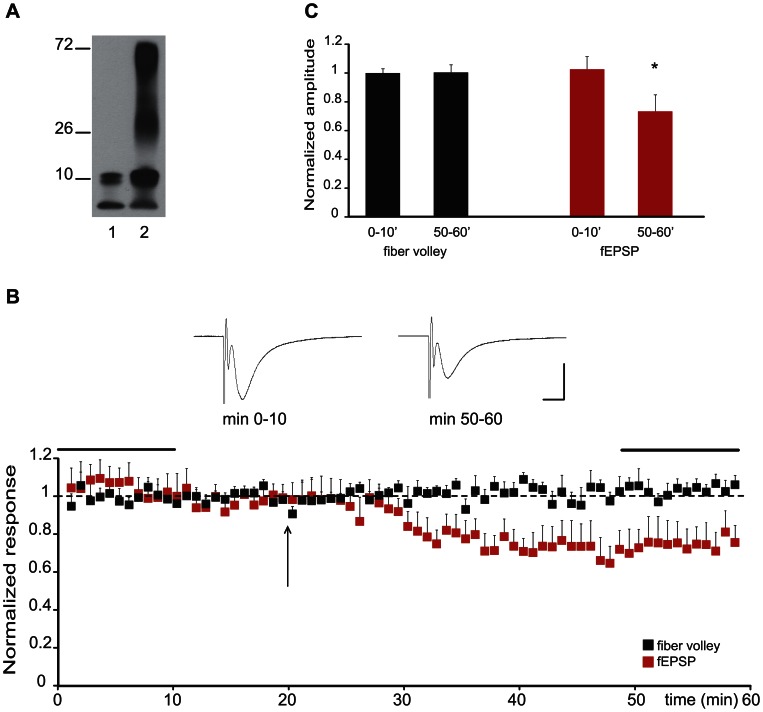
Aβ perfusion causes depression of evoked field potentials. (A) Western blot of an Aβ oligomer preparation. Lanes illustrate protein separation before (1) and after (2) induction of Aβ oligomerization. Numbers on the left indicate protein size in kD. (B) Recording of Aβ-induced changes in the slope of fEPSPs and fiber volley amplitude. Aβ was added after recording a 20 min baseline (black arrow). Averaged traces taken during baseline recording and following Aβ oligomer perfusion (horizontal black bars in the scatter blot) are shown above the graph. Scale bars: horizontal: 10 ms; vertical 0.5 mV. (C) Quantification of the experiment shown in B. Acute Aβ perfusion leads to a fast decrease of evoked extracellular field potentials without affecting fiber volley amplitudes. (*p<0.01, n = 8).

**Figure 2:**
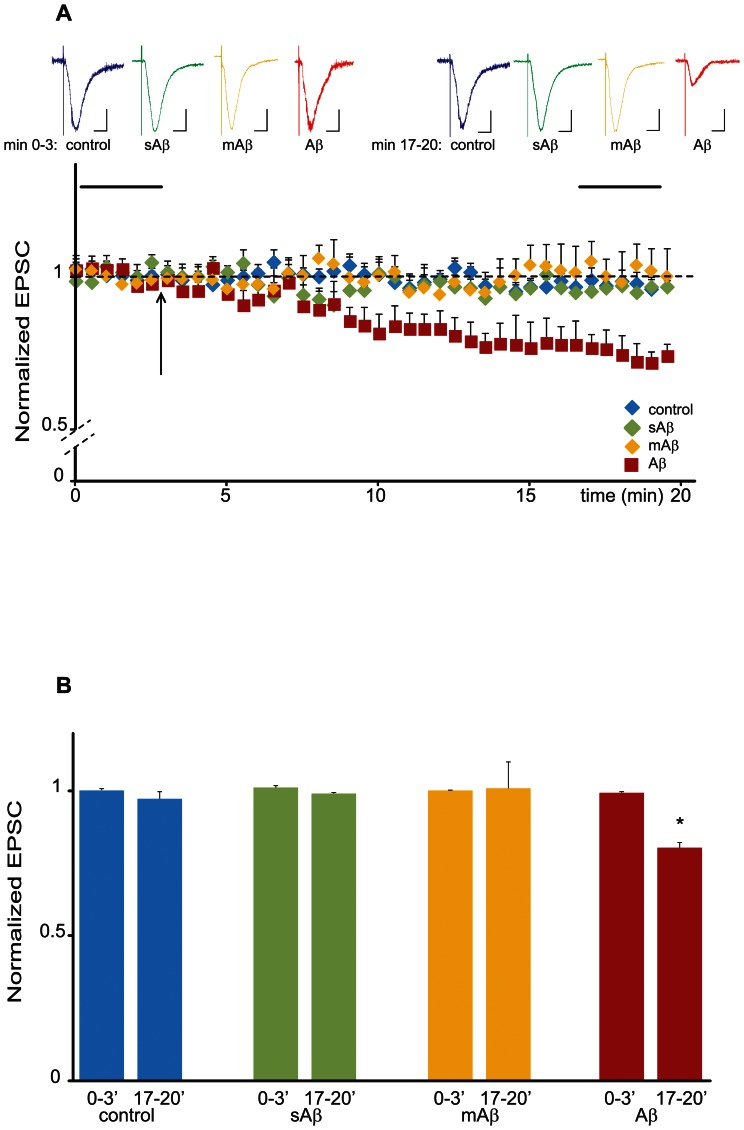
Aβ depresses synaptic transmission. (A) Changes in normalized EPSC amplitudes plotted against time in presence of Aβ oligomers, vehicle, scrambled and monomeric Aβ. Neurons were patched and held at −60 mV and responses were evoked throughout the experiment. After baseline recording, Aβ (red trace), vehicle (blue trace), scrambled Aβ (sAβ, green trace) or monomeric Aβ (mAβ, yellow trace) were applied to the bath solution. The arrow indicates when either the peptides or the vehicle solution was bath applied. Averaged traces taken during baseline recording and 15 min after peptide or vehicle application (horizontal black bars in the scatter blot) are shown above the graph. Scale bars: horizontal 0.1 s; vertical 10 pA. (B) Quantification of the experiment shown in A. Acute Aβ perfusion leads to a fast induction of synaptic depression. (*p<0.01, n = 6).

We therefore acutely perfused oligomeric Aβ (0.1 µM, Fig. 1a) and analyzed its effects on evoked field potentials. After having established a stable baseline recording for 20 min, we perfused the slice cultures with oligomeric Aβ for 20 min. As seen in Figure 1 b and c, Aβ oligomers induced a significant synaptic depression within 10 min of exposure. We did not observe a difference in the fiber volley amplitude upon Aβ perfusion (Fig. 1 b and c), indicating that the acute Aβ-dependent synaptic depression does not act presynaptically. To further analyze the synaptic mechanisms of acute Aβ induced synaptic depression, we recorded evoked basal synaptic transmission in CA1 neurons in whole-cell patch clamp configuration (−60 mV holding potential) by stimulating the Schaffer collaterals. After 3 min of baseline recording, we perfused slices with oligomeric Aβ. As seen in Figure j, we observed a statistically significant synaptic depression within 10–15 minutes after Aβ application. This depression was specific for Aβ oligomers, since the scrambled form of Aβ (randomized Aβ amino acid sequence peptide), the monomeric Aβ as well as vehicle alone had no effect on basal synaptic transmission (Fig. 2a and 2b).

**Figure 3:**
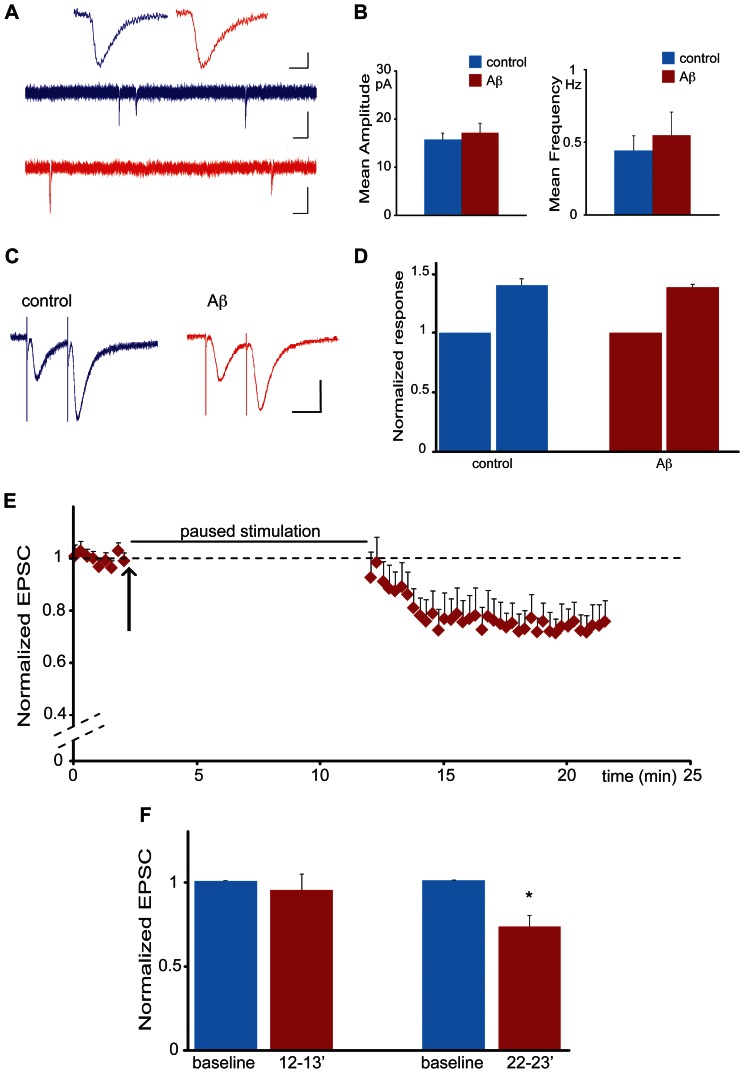
Aβ induced synaptic depression is dependent on synaptic stimulation. (A) Miniature EPSCs Upper traces: average of miniature events after perfusion of control (blue) or Aβ (red). Scale bars: horizontal, 5 ms; vertical 5 pA. Middle and lower traces: sample of a four seconds recording of miniature EPSC activity following perfusion of control (blue) or Aβ oligomers (red). Scale bars: horizontal 0.2 s; vertical 10 pA. (B) Perfusion of Aβ oligomers (red) does not change the amplitude (left) or the frequency (right) of recorded miniature events when compared to vehicle control (blue) (n = 20 for each condition). (C) EPSC recordings obtained by applying a paired pulse facilitation protocol under control conditions (blue trace) and after Aβ oligomers perfusion (red trace). Inter pulse interval was 30 ms. Scale bars: horizontal, 20 ms; vertical 20 pA. (D) Quantification of the experiment shown in C. Perfusion of Aβ oligomers (red) does not affect paired-pulse facilitation when compared to control (blue). The response to the second pulse was normalized by the response to the first pulse (n = 10 for each condition). (E) Aβ induced synaptic depression is dependent on synaptic stimulation. Normalized EPSCs from CA1 neurons are shown. After baseline recordings, Aβ oligomers were added to the bath solution (black arrow) and the stimulation protocol paused for 10 minutes (represented by the black bar) before being resumed for another 10 min. (F) Quantification of the experiment shown in E. Bars indicate responses recorded during baseline (blue) and in presence of Aβ (red) at different time intervals: minute 12–13 averages responses recorded immediately after stimulation was resumed; minute 22–23 averages responses 10 minutes after stimulation was resumed (i.e. 19–20 minutes after the start of Aβ perfusion) (*p<0.05, n = 8).

To further characterize the basis of Aβ induced changes in synaptic transmission, we recorded synaptic miniature events in the presence of Aβ. We perfused slice cultures for 20 min with Aβ or vehicle, added TTX for 10 min and recorded miniature events (Fig. 3a). As seen in Figure 3b, we observed no changes in miniature amplitudes or frequency in the presence of Aβ and TTX. The unchanged miniature frequency is consistent with our data showing that Aβ does not affect fiber-volley amplitudes (Fig. 1b and c) or paired-pulse facilitation (Figure 3c and d), indicating that the presynaptic site is not involved in acute Aβ dependent synaptic depression. However, the lack of change in miniature amplitudes while having previously observed synaptic depression in field recordings (Fig. 1b) as well as whole-cell patch-clamp recordings (Fig. 2a), suggested that Aβ mediated synaptic depression is dependent on synaptic stimulation. To test this hypothesis, we repeated our experiment seen in Fig. 2a, but paused external stimulation during the first 10 min of Aβ perfusion. Resuming stimulation after 10 min Aβ perfusion revealed no synaptic depression for the first minutes, indicating that Aβ induced depression is dependent on synaptic stimulation. Consequently, 5 min after resuming stimulation, synaptic transmission became significantly reduced (Figure 3e and f; the steeper induction curve of depression in comparison to Fig. 2a is most likely due to Aβ already being present at the synapse when stimulation is resumed, i.e. slices are already perfused for 10 min with Aβ). Taken together, we conclude that Aβ can rapidly induce synaptic depression, but only in the presence of sufficient synaptic activity.

**Figure 4:**
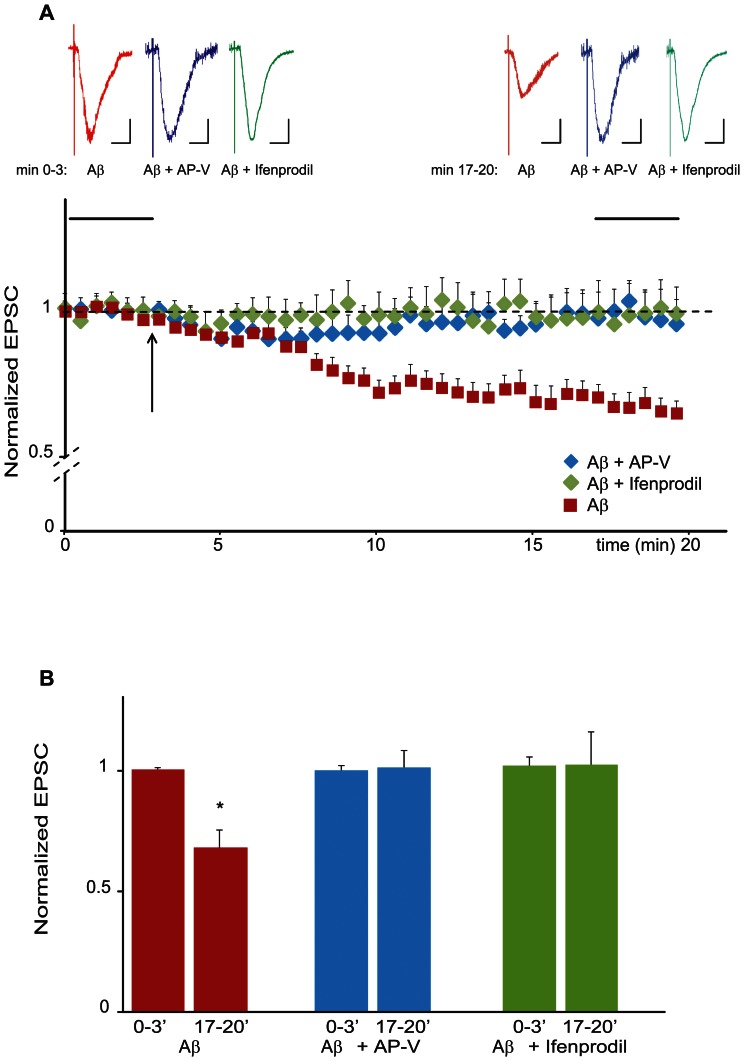
Aβ induced synaptic depression is mediated by NMDA-receptor activation. (A) Aβ dependent changes in normalized EPSC amplitudes plotted against time in the presence of the NMDA-receptor antagonists APV and Ifenprodil. APV or Ifenprodil were bath applied prior to the beginning of the experiments. Neurons were patched and held at −60 mV; responses were evoked throughout the experiment. After baseline recording, Aβ was applied to the bath (black arrow). Averaged traces taken during baseline recording and 15 min after Aβ application (horizontal black bars in the scatter blot) are shown above the graph. Scale bars: horizontal 0.1 s; vertical 10 pA. (B) Aβ-dependent synaptic depression is mediated by NMDA-receptor activation. Quantification of the experiment shown in A. Bar diagram showing changes to the normalized mean EPSC due to Aβ (red), Aβ+AP-V (blue) and Aβ+Ifenprodil (green) perfusion. (*p<0.01, n = 7).

Given that synaptic activity was necessary for the acute effect of Aβ on synaptic depression, we wondered whether this depression would be sensitive to NMDA-receptor (NMDA-R) activation as it was shown with chronic APP expression [14]. We therefore added the competitive NMDA-R specific antagonist APV to the bath solution 10 min prior to recording. Interestingly, APV completely blocked acute Aβ dependent synaptic depression (Fig. 4). This result was surprising, since the NMDA-R is known to be blocked by magnesium ions when the cell is at resting potential or, as in our case, clamped at −60 mV resting potential. To further analyze which of the two NMDA-R subunits (GluN2A or GluN2B) would be responsible for Aβ dependent synaptic depression, we repeated the experiment in the presence of Ifenprodil, a GluN2B specific inhibitor. As shown in Figure 4 Ifenprodil, like APV, blocked Aβ dependent synaptic depression. Taken together, we could show that the acute perfusion of oligomeric Aβ leads to an activity dependent synaptic depression involving GluN2B containing NMDA-Rs, even if the cell is clamped at resting potential and therefore preventing ion influx through NMDA-receptors.

### Acute Aβ Dependent Synaptic Depression does not Require NMDA-receptor Dependent Ion Flux

Given that Aβ induces NMDA-R dependent synaptic depression at a holding potential of −60 mV, we speculated that oligomeric Aβ could act by loosening the magnesium block that normally prevents NMDA-R dependent transmission at resting potential, i.e. Aβ would increase NMDA-R dependent synaptic currents at resting potential.

**Figure 5:**
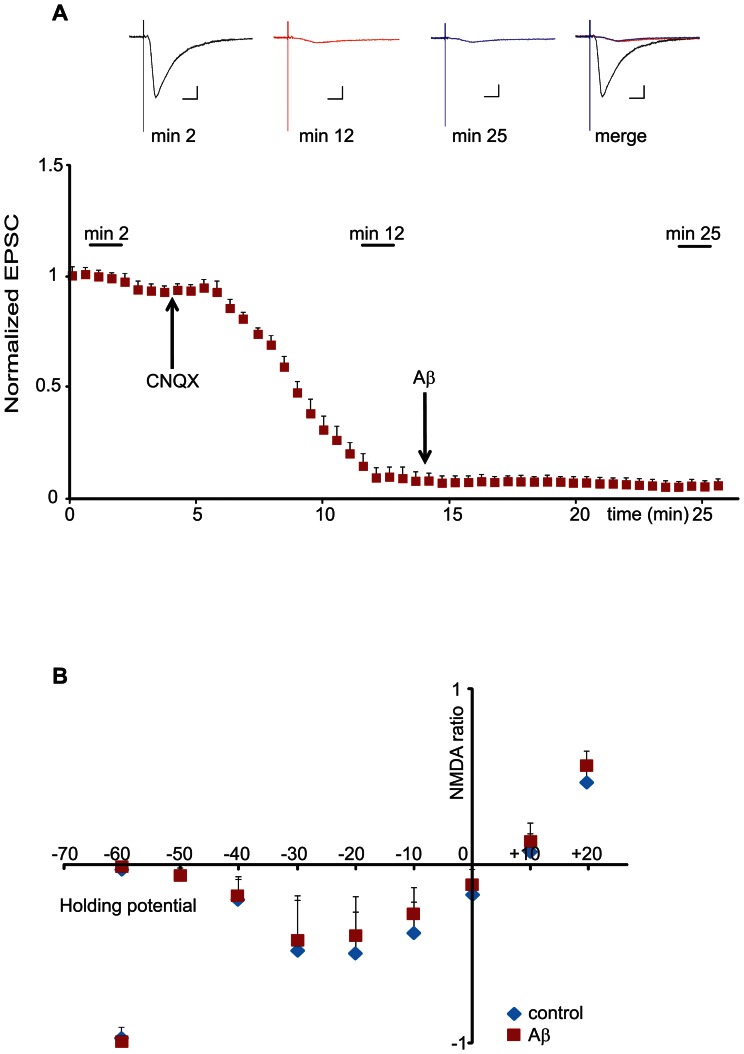
Aβ has no effect on NMDA-receptor mediated ion conductance. (A) Normalized EPSC amplitudes plotted against time in presence of CNQX and Aβ. Neurons were patched and held at −60 mV and responses were evoked throughout the duration of the experiments. Following baseline recording, CNQX was applied to the bath to block AMPA-receptor dependent currents (arrow on the left). Following the sharp decrease in the EPSC amplitude, Aβ oligomers were applied (arrow on the right). Averaged traces taken during baseline recording (min 2), after CNQX application (min 12) and after Aβ application (min 25) are shown above the graph. There is no evident difference between currents recorded immediately before Aβ application and currents recorded 10 minutes following Aβ application (n = 6). Color legend: black: currents recorded before CNQX application; red: currents recorded before Aβ application; blue: currents recorded after Aβ application. Scale bars: horizontal 0.1 s; vertical 10 pA. (B) Normalized NMDA-receptor I/V curve recorded in presence or absence of Aβ oligomers. Evoked synaptic responses of CA1 pyramidal neurons were recorded at −60 mV. Next, CNQX was bath applied to block AMPA-receptor currents. Following AMPA-receptor blockade, either vehicle or Aβ was bath applied. 15 min after Aβ application, evoked EPSCs (now dependent on NMDA-receptor responses) were recorded at different holding potentials (steps of 10 mV increments from −60 to +20 mV holding potential). Calculating the ratio of NMDA-receptor responses vs. the initial AMPA-receptor response was used to normalize the NMDA-receptor currents from different experiments. No noticeable difference in NMDA-receptor responses was found between neurons treated with vehicle vs. Aβ oligomers (n = 5 per group).

To analyze changes in NMDA-R dependent synaptic transmission, we recorded isolated NMDA-R responses by employing the AMPA/Kainate-receptor inhibitor CNQX. As seen in Figure 5a, CNQX completely blocks AMPA-receptor dependent transmission. After AMPA-receptor dependent transmission was blocked, we perfused oligomeric Aβ for 15 min and recorded evoked synaptic responses. However, we did not observe any increase in NMDA-R mediated synaptic transmission, as we would have expected if Aβ could increase ion leakage through the NMDA-receptor.

Given this result, we wondered whether small local depolarization in dendritic spines at the moment of AMPA-receptor activation would be sufficient to affect NMDA-receptor dependent synaptic transmission in the presence of Aβ, i.e. Aβ would weaken the magnesium block only under depolarized conditions. Since the sensitivity of our experimental setup in Figure 5a would be insufficient to detect these changes, we decided to compare evoked NMDA-R currents at different holding potentials with and without oligomeric Aβ. We first recorded synaptic AMPA-receptor dependent transmission without exposure to Aβ, followed by the perfusion of Aβ for 15 minutes and the addition of the AMPA/Kainate-receptor inhibitor CNQX thereafter. With AMPA-receptors blocked, the remaining NMDA-receptor dependent synaptic transmission was recorded at different holding potentials (−60 to +20 mV in 10 mV increments). Each of the recorded NMDA-receptor responses was then normalized to the correspondent AMPA-receptor dependent response obtained during the first 3 minutes of recording without Aβ or CNQX. As seen in Figure 5b, Aβ does not change NMDA-R dependent synaptic transmission at different holding potentials, indicating that Aβ does not act by loosening the magnesium block in NMDA-Rs. Furthermore, we did not observe a change in NMDA-R dependent transmission when the neuron was clamped at +10 mV or +20 mV, indicating that Aβ does not change NMDA-R dependent synaptic transmission in the absence of magnesium block. Taken together, we concluded that oligomeric Aβ does not affect the ion flux via the opening of NMDA-Rs.

**Figure 6:**
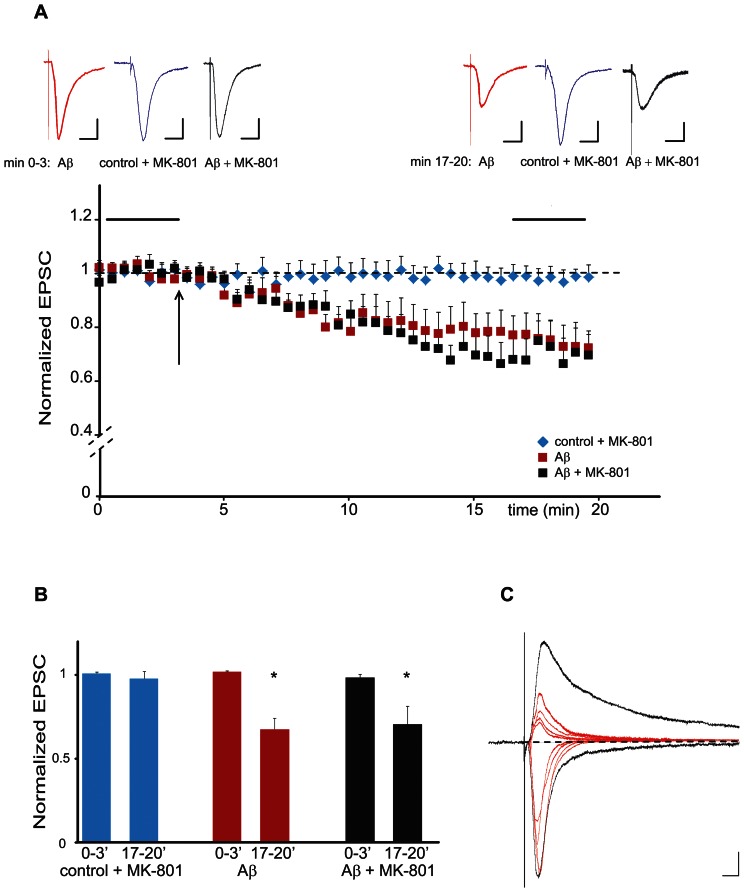
The NMDA-receptor channel blocker MK-801 does not affect Aβ-induced synaptic depression. (A) Aβ dependent changes in normalized EPSC amplitudes plotted against time in the presence of the NMDA-receptor channel blocker MK-801. The use-dependent NMDA-receptor blocker MK-801 was added to the slice culture medium 12 h before slices were transferred to the MK-801 containing bath solution. Neurons were patched and held at −60 mV and responses were evoked throughout the experiment. After baseline recording, either Aβ or vehicle was applied to the bath solution (black arrow). Averaged traces taken during baseline recording and 15 min after Aβ application (horizontal black bars in the scatter blot) are shown above the graph. Color legend: red: Aβ; blue: control in presence of MK-801; black: Aβ+MK-801. Scale bars: horizontal 0.1 s; vertical 10 pA. (B) Aβ dependent synaptic depression is not blocked by MK-801. Quantification of the experiment shown in A. Bar diagram showing changes of the normalized mean EPSC due to the presence of control+MK-801 (blue), Aβ (red) and Aβ+MK-801 (black). (*p<0.01, n = 6 for Aβ; p<0.01, n = 13 for Aβ+MK-801). (C) Overnight exposure to MK-801 is able to completely block NMDA-receptor dependent synaptic transmission. Evoked EPSCs recorded in absence (black traces) and after 12 hours MK-801 incubation (red traces). Black traces were recorded from a neuron pre-incubated with vehicle solution, while red traces were obtained from four different neurons that had been pre-incubated for 12 hours with MK-801. Recordings were performed at −60 mV (to record AMPA-receptor currents) and +40 mV (to record NMDA- and AMPA-receptor mediated currents). Traces are based on an average of 60 samples. Scale bars: horizontal 20 ms; vertical 10 pA.

So far, it appears that synaptic activity (Fig. 3e) and GluN2B containing NMDA-R activation (Fig. 4) are necessary for the effect of Aβ on synaptic depression, but that Aβ is not changing the ion flux through the NMDA-Rs. Furthermore, ion flux through the NMDA-receptor is virtually absent at −60 mV resting potential (Fig. 5b). These findings led us to hypothesize that Aβ in combination with synaptic activity is activating a NMDA-R dependent intracellular signaling pathway even in the absence of ion flux through the NMDA-R, i.e. the NMDA-R would act like a metabotropic receptor. To test this hypothesis, we made use of MK-801, a use dependent NMDA-R ion channel blocker. Different from APV, which interferes with the binding of glutamate to the NMDA-R, MK-801 binds within the pore of NMDA-Rs. We therefore expected no effect on Aβ dependent synaptic depression if NMDA-Rs were to act in a metabotropic like fashion. We incubated slice cultures for 12 hours with MK-801 prior to recording the effects of Aβ on synaptic transmission. As seen in Fig. 6a and b, Aβ was still able to induce synaptic depression in presence of MK-801. To exclude that 12 hours of MK-801 incubation would be insufficient to block NMDA-receptor dependent synaptic transmission, we recorded in a separate experiment evoked synaptic currents at −60 mV and +40 mV holding potential after MK-801 incubation. As shown in Fig. 6c, at positive holding potentials, neurons treated with MK-801 show a complete block of NMDA-R responses at +40 mV compared to untreated controls (the remaining response is composed of the AMPA-R response). In summary, we could show that acute Aβ exposure leads in the presence of MK-801 to the induction of synaptic depression. This depression engages a use-dependent but ion-flux independent activation of GluN2B containing NMDA-Rs. Our results therefore point toward a new metabotropic-like mechanism in NMDA-R dependent signal transduction.

## Discussion

Amyloid-beta is considered to be one of the most prominent factors in the development of Alzheimer’s disease. Transgenic AD animal models and in-vitro models with chronic over-expression of Aβ have broadened our understanding of the physiological changes occurring during Alzheimer disease. However, in all of these models, it is hard to distinguish whether the observed effects are directly caused by Aβ or whether they are reflecting the neurons counter-response to the primary Aβ insult. To shed light on this question, we acutely perfused hippocampal slice cultures with oligomeric Aβ. Within 20 minutes of Aβ perfusion we observed a NMDA-R dependent synaptic depression. To our surprise, the involved NMDA-receptors act in this scenario like metabotropic receptors, i.e. an NMDA-receptor mediated ion-flux is not necessary for Aβ induced synaptic depression.

### The NMDA-receptor Acting as a Metabotropic Receptor

Our results suggest that NMDA-receptors, in the presence of Aβ, can act as metabotropic receptors; thus, upon ligand binding, these receptors can activate intracellular signaling cascades in the absence of ion flux. While this concept of NMDA-receptor activity appears to be rather new, it is however not unprecedented. Several laboratories described intracellular effects of glutamate binding on NMDA-receptors in the absence of ion flux. In 2001, the group of Gary Westbrook showed that the Tyrosine residues in GluN1/GluN2A containing NMDA-receptors are dephosphorylated in a use-dependent fashion without involving ion flux [15]. This dephosphorylation would ultimately lead to an endocytosis of synaptic NMDA-receptors. The group of Michael Salter found that extracellular glycine binding to the NMDA-receptor primes the receptor for endocytosis by recruiting intracellular AP2 to the receptor complex. Again, the effects were independent of ion flux through the NMDA-receptor [16]. The group of Roberto Malinow has shown that the exchange of GluN2B by GluN2A in the CA1-CA3 hippocampal synapse is driven by spontaneously released glutamate, even in the absence of synaptic NMDA-receptor dependent currents [17], indicating that the NMDA-receptor might act in a metabotropic fashion. Finally, a more recent publication by the group of John Wang described the activation of extracellular signal-regulated protein kinase (ERK) through the co-activation of metabotropic glutamate receptor 5 (mGluR5) and NMDA-receptors [18]. Interestingly, the authors were able to block ERK activation by applying the NMDA-receptor antagonist APV (APV prevents glutamate binding and thereby activation of NMDA-receptors), while the non-competitive NMDA-receptor channel blocker MK-801, had no effect on ERK activation.

Our results furthermore, shed new light on the AD medication memantine. Memantine, like MK-801, is an open channel blocker targeting the ion pore of NMDA-receptors and thereby reducing the ion flux [19]. Given that oligomeric Aβ would activate the NMDA-receptor in a metabotropic-like fashion, memantine should have no or very limited effects on Aβ induced synaptic depression. Our results could therefore contribute in explaining why memantine was ineffective in treating early stages of AD [20].

### Aβ Induced Synaptic Depression

Our data support the notion that while Aβ induced LTD-like synaptic depression is dependent on NMDA-receptor activation (i.e. ligand binding; Fig. 4), it appears to be independent of calcium flux through the NMDA-receptor (Fig. 6). At this moment, we can only speculate how the NMDA-receptor, acting in a metabotropic fashion, might induce synaptic depression.

Recently, several groups have shown that Aβ induced synaptic depression by facilitating NMDA-receptor dependent LTD or mGluR dependent LTD [9], [21]. Furthermore, blocking the NMDA-receptor with the competitive antagonist APV, also blocks Aβ dependent induction of synaptic depression [14], [22], while the chronic expression of the amyloid-precursor protein was also able to occlude mGluR dependent LTD induction [14]. All of these results suggest that Aβ can mimic LTD-like pathways, leading to the chronic induction of synaptic depression. In general, the literature suggests that NMDA-receptor dependent LTD and mGluR-dependent LTD are largely based on different cellular mechanisms and induction pathways [23]. However, it is also known that mGluR5 is able to modulate the outcome of NMDA-receptor mediated LTD (e.g. [24]). Aβ can act as an extracellular scaffold for mGluR5, leading to an increased clustering of mGluR5 in synapses accompanied by an increased concentration of intracellular calcium [24]. Hence, it is possible that Aβ dependent synaptic depression involves the co-activation of NMDA-receptors and mGluR5. Of interest is hereby the aforementioned study by Yang et al., which showed that the activation of mGluR5 and NMDA-receptors in the absence of NMDA-receptor dependent ion flux is able to induce ERK5 activation [18], [25]. ERK activation on the other hand is necessary for the induction of mGluR dependent LTD [25]. Beside mGluR5, Aβ was described to bind the alpha7 nicotinic acetylcholine receptor [26], a receptor that was later on shown to activate the striatal-enriched tyrosine phosphatase (STEP) [27]. Again, STEP appears to be necessary for the induction of mGluR-dependent LTD. Through its effect on NMDA-receptor and AMPA-receptor dephosphorylation and subsequent receptor internalization, STEP was described as a more general modulator of synaptic plasticity [28]. Finally, Aβ was described to bind EphB2, a receptor that directly interacts and modulates NMDA-receptor surface expression and activity [29]. However, the involvement of EphB2 in synaptic plasticity remains to be characterized. Taken together, Aβ is able to interact with several receptors, some of them, like mGluR5 and alpha7 nicotinic receptor, are directly involved in regulating synaptic plasticity. Which of these receptors together with the NMDA-receptor is responsible for the effects of Aβ on synaptic depression remains a topic for future investigation.

## Methods

### Aβ Preparation

Aβ 1–42 (Bachem) was prepared according to Barghorn et al [30]. Oligomeric Aβ was prepared fresh every three days and stored at 4°C when not used. As control, we prepared the same solution but with scrambled Aβ (Covance) or without any peptide at all. We also performed an additional control, employing non-oligomerized monomeric Aβ. To analyze the formation of oligomeric Aβ, 100 ng of Aβ were loaded on a 10–20% discontinuous gradient Tris-Tricine gel. Western blot analysis was performed using the 6E10 anti-Aβ antibody (Millipore).

### Slice Preparation

Hippocampal slices were prepared from p7 rat pups as previously described in [31], [32] and maintained in culture for 5 to 7 days. For electrophysiological recording, slices were transferred to a submerged recording chamber maintained at 32°C.

### Electrophysiological Protocols

Whole-cell recordings were obtained from CA1 pyramidal neurons; recordings were performed using a MultiClamp700B amplifier (Axon Instruments), and data was acquired using Clampex 10.2 software (Axon Instruments). The recording chamber was perfused with ACSF (119 mM NaCl, 2.5 mM KCl, 26 mM NaHCO3, 1 mM NaH2PO4, 11 mM glucose) containing 0.1 mM Picrotoxin (Sigma), 4 mM CaCl_2_, 4 mM MgCl_2_ and 4 mM 2-chloroadenosine (Sigma), bubbled with a Carbogen mix (5% CO_2_, 95% O_2_). Patch pipettes (4–6 MΩ) were filled with internal solution containing: 115 mM cesium methanesulfonate, 20 mM CsCl, 10 mM HEPES, 2.5 mM MgCl_2_, 4 mM Na_2_ATP, 0.4 mM Na_3_GTP, 10 mM sodium phosphocreatine, 0.6 mM EGTA, at pH 7.25 and 290 mosm. Cells were patched under visual guidance and clamped at −60 mV unless otherwise specified.

EPSCs were evoked by stimulating the Schaffer collaterals at 0.2 Hz through two bipolar tungsten electrodes, connected to a stimulus isolation unit (A.M.P.I.). The electrodes were positioned ∼150 and ∼250 µm away from the recording site. This setting allowed us to stimulate two independent synaptic inputs; results from both pathways were then averaged and counted as n = 1. Given that slices were only used once for recordings, n reflects the number of neurons as well as the number of slices (usually from 4–6 different animals per experiment). All recordings were performed at 32°C. Baseline recordings lasted for 3 to 5 minutes, following perfusion of either Aβ or the control vehicle. After application of drugs or Aβ, neurons were usually recorded for a minimum of 20 minutes and the results compared to the baseline recordings.

For the I/V profile, neurons were patched and held at −60 mV and a baseline of 3 minutes was recorded. Following baseline recording, CNQX was then bath applied for 5 min in order to block AMPA receptor dependent transmission (AMPA receptor dependent currents were no longer observed). Next, either Aβ or the control vehicle was bath applied for 20 minutes. Neurons were then stepwise depolarized from −60 mV to +20 mV in order to determine the amplitude of NMDA receptor responses recorded at each holding potential. We recorded a minimum of 2 min per stepping potential. Responses from Aβ–treated and untreated neurons were analyzed and displayed as the ratio of the NMDA response at a specific holding potential normalized by the corresponding AMPA receptor response recorded before at −60 mV.

For the recordings of miniature events, either Aβ or vehicle was bath applied prior to the beginning of the experiment. As above, 20 minutes were allowed for Aβ or vehicle to take effect, followed by TTX (1 µM) bath application. 5 minutes after drug application, neurons were patched and miniature events recorded for a minimum of 10 minutes. Miniature events were then analyzed off-line employing the Clampfit 10.2 software.

### Drugs

Drugs were usually applied to the bath solution prior to the beginning of the experiment (unless otherwise specified). We employed the AMPA-receptor antagonist CNQX (Tocris, 20 µM), the NMDA-receptor specific antagonists AP-V (TOCRIS, 100 µM) and Ifenprodil (Abcam Biochemicals, 10 µM). Also, in some experiments we used the NMDA-receptor blocker MK-801 (Tocris, 25 µM). MK-801 was added to the cultured slices the night before and MK-801 was present at the same concentration during the recording. For miniature events analysis, experiments were conducted in presence of the Na^+^ channels blocker TTX (Alomone labs, 1 µM).

### Statistical Analysis

Results are displayed as bar diagrams or scatter graphs, representing the experimental mean; the error bars represent the standard error. For statistical analysis we used the non-parametric Wilcoxon-Mann-Whitney test.

### Ethics Statement

The animal protocol was approved by the “Comité de déontologie de l'expérimentation sur les animaux» (CDEA) of the University Montreal. (Permit Number: 12–185).

Note added in proof: While this manuscript was under review, the group of Roberto Malinow employing a different experimental approach reported comparable results with similar conclusions [33].
